# Zirconium ferrite incorporated zeolitic imidazolate framework-8: a suitable photocatalyst for degradation of dopamine and sulfamethoxazole in aqueous solution†

**DOI:** 10.1039/d3ra01055d

**Published:** 2023-03-23

**Authors:** Adewale Adewuyi, Olaoluwa A. Ogunkunle, Rotimi A. Oderinde

**Affiliations:** a Department of Chemical Sciences, Faculty of Natural Sciences, Redeemer's University Ede Osun State Nigeria walexy62@yahoo.com +2348035826679; b Department of Chemistry, University of Cambridge Lensfield Road Cambridge CB2 1EW UK; c Department of Chemistry, Obafemi Awolowo University Ile-Ife Nigeria; d Department of Chemistry, Faculty of Science, University of Ibadan Ibadan Oyo State Nigeria

## Abstract

The complete removal of pharmaceutical wastes from polluted water systems is a global challenge. Therefore, this study incorporates zirconium ferrite (ZrFe_2_O_4_) into zeolitic imidazolate framework-8 (ZIF-8) to form ZrFe_2_O_4_@ZIF-8. The ZrFe_2_O_4_@ZIF-8 is a photocatalyst for removing dopamine (DOP) and sulfamethoxazole (SMX) from an aqueous solution. The scanning electron micrograph revealed the surfaces of ZrFe_2_O_4_ and ZrFe_2_O_4_@ZIF-8 to be heterogeneous with irregularly shaped and sized particles. The transmission electron micrograph (TEM) images of ZrFe_2_O_4_ and ZrFe_2_O_4_@ZIF-8 showed an average particle size of 24.32 nm and 32.41 nm, respectively, with a bandgap of 2.10 eV (ZrFe_2_O_4_@ZIF-8) and 2.05 eV (ZrFe_2_O_4_). ZrFe_2_O_4_@ZIF-8 exhibited a better degradation capacity towards DOP and SMX than ZrFe_2_O_4_. ZrFe_2_O_4_@ZIF-8 expressed a complete (100%) degradation of DOP and SMX during the photodegradation process. Interestingly, the process involved both adsorption and photocatalytic degradation simultaneously. ZrFe_2_O_4_@ZIF-8 demonstrated high stability with a consistent regeneration capacity of 98.40% for DOP and 94.00% for SMX at the 10^th^ cycle of treatment in a process described by pseudo-first-order kinetics. The study revealed ZrFe_2_O_4_@ZIF-8 as a promising photocatalyst for the purification of DOP and SMX-contaminated water systems.

## Abstract 2

The complete removal of pharmaceutical wastes from polluted water systems is a global challenge.

## Graphical abstract

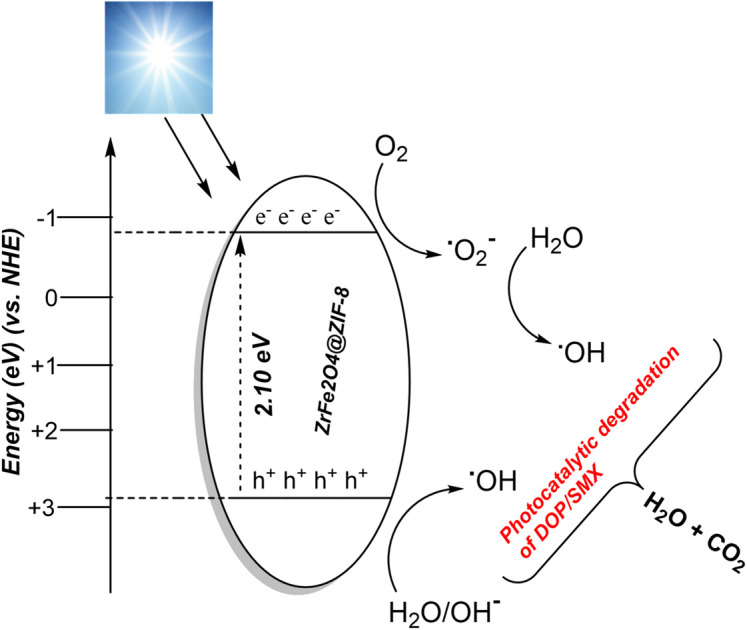

## 1 Introduction

The inability to completely remove pharmaceutical wastes from drinking water sources is a global problem. Different classes of pharmaceutical wastes have been detected in surface and underground water systems, wastewater treatment discharges and domestic wastewater.^1–4^ The pharmaceutical wastes in water are toxic emerging contaminants because of their capacity to become a threat to human beings and aquatic life. Their presence in water is undesired, and it is crucial to eliminate them. Therefore, this study focuses on developing a means to remove toxic pharmaceutical contaminants in water. Dopamine (DOP) and sulfamethoxazole (SMX) are examples of undesired pharmaceutical contaminants detected in water.^5,6^ The continuous use of DOP in chemical synthesis has contributed immensely to its presence in the laboratory and industrial effluents. DOP and SMX are readily available and are easily purchased without a prescription in most developing countries.

The continuous use of SMX in treating ailments in human and animal husbandry has aided its frequent occurrence in drinking water sources. SMX is a known antibiotic for treating infections.^7–9^ SMX is very stable (thermal and photostability) in the environment, which gives it a prolonged presence when it gets into drinking water sources, making it possible for it to be transported from one point to another. The persistence of SMX in the environment has contributed to the emergence of drug-resistant strains of pathogenic organisms.^9,10^ The emergence of drug-resistant pathogens is a serious global challenge with many concerns.^11^ One of the ways to address these concerns is to develop a means for the complete removal of active drug species in water systems. Previous studies^12,13^ reported SMX in surface water (0.94 μg L^−1^), effluent discharges (24.81 μg L^−1^) and potable water (12.00 μg L^−1^). Most studies have reported the concentration of SMX in the environmental water system to vary from ng L^−1^ to μg L^−1^.^14–16^. Even though SMX remains one of the early detected antibiotics in water, its complete removal in water is still a challenge.

Besides being a neuromodulator for treating conditions such as Parkinson's disease, DOP is used for several syntheses. It has been used to prepare nanocomposites^17–19^ and other improved products.^20,21^ When used during synthesis, they are generated into laboratory waste and discarded in laboratory effluent. Many biochemical laboratories in tertiary institutions and research institutes use DOP during practical sessions in which they get into wastewater generated. Most of these laboratories need more capacity to remove DOP in wastewater generated entirely. Furthermore, the presence of DOP biomarkers has been reported in a wastewater-based epidemiology study.^22^ Other studies suggest its presence in the environment.^23–25^ When present in an environmental water system and under certain environmental factors, both DOP and SMX could metamorphose into new compounds which may be hazardous to humans and the environment suggesting their immediate removal.

Many methods have been reported to remove SMX in water systems.^26,27^ Recently, a study reported the synthesis of a ternary LTO/CN/AgI nanohybrid catalyst with multicharged transfer channels for the degradation of SMX.^28^ The catalyst demonstrated a high kinetic rate constant of 0.25776 min^−1^ for the degradation process with an insight at the molecular level. Some authors combined hydrothermal and photodeposition as methods for the preparing Ag/g-C_3_N_4_ (CN)/Bi_3_TaO_7_ (BTO) as photocatalyst for the degradation of SMX under the influence of visible-light. Although there was an improved performance but there was no complete removal (100%) of the SMX in solution.^29^ Furthermore, Co doped ZnO nanorods and other some other potential photocatalysts have shown capacity as efficient catalyst for water purification^30–32^ while AgNbO_3_ corroborated this fact under visible light with an impressive performance with without still attaining complete removal of SMX in solution.^33^

Unfortunately, these methods have shown some drawbacks that could be more improvable. The major drawback of these methods is the inability to remove SMX from contaminated water systems completely. On the other hand, there are limited studies on removing DOP in water. It is crucial to investigate the removal of DOP from aqueous solution due to its frequent use and entrance into the environment. Photocatalysis remains an effective method for removing organic molecules in polluted water systems.^34–36^ The photocatalysis process involves using a photocatalyst to promote the oxidation of organic molecules in water to CO_2_ and H_2_O. Some studies have reported using nanoparticles, such as semiconductors, to remove organic molecules in water.^37–39^ Moreover, such semiconductors can also be photocatalysts for photodegrading organic contaminants in water. Sadly, some semiconductors are expensive or limited in their activity in the visible light region. It is essential to use photocatalysts with efficient action in the visible light region to reduce process costs since visible light is freely available. Therefore, this study suggests zirconium ferrite (ZrFe_2_O_4_) as an effective photocatalyst in the visible light region.

ZrFe_2_O_4_ is of interest because of its unique properties, such as small size, thermal stability, optical properties, and electrical properties. Unfortunately, particles of ZrFe_2_O_4_ aggregate, which causes recombination limiting its photocatalytic activity. Therefore, this study proposes the inclusion of ZrFe_2_O_4_ in a metal–organic framework, zeolitic imidazolate framework-8 (ZIF-8), forming ZrFe_2_O_4_@ZIF-8 to circumvent the challenge. In the structure of ZrFe_2_O_4_@ZIF-8, ZIF-8 serves as a carbon source inhibiting the aggregation of ZrFe_2_O_4_ particles and enhancing its recovery from solution. Currently, there are limited report on the photodegradation of DOP and SMX by ZrFe_2_O_4_@ZIF-8. The current study, therefore, aimed at achieving the complete removal of DOP and SMX in contaminated water systems using ZrFe_2_O_4_@ZIF-8.

## 2 Experimental

### 2.1. Materials

Zirconium oxychloride octahydrate (ZrOCl_2_·8H_2_O), sodium hydroxide (NaOH), zinc nitrate hexahydrate (Zn(NO_3_)_2_·6H_2_O), 2-methylimidazole (C_4_H_6_N_2_), polyvinylpolypyrrolidone (PVP), iron(iii) chloride hexahydrate (FeCl_3_·6H_2_O), ethanol (C_2_H_5_OH), chloroform (CH), ammonium oxalate (AO), hydrochloric acid (HCl), DOP, SMX, isopropyl alcohol (IPA), and other chemicals were ordered from Aldrich Chemical Co., England.

### 2.2. Synthesis of ZrFe_2_O_4_ particles

ZrFe_2_O_4_ was prepared by mixing solutions of ZrOCl_2_·8H_2_O (0.2 M) and FeCl_3_·6H_2_O (0.4 M) in a beaker (1 L) for 60 min in the presence of PVP. The mixture's temperature was gradually raised to 80 °C, and pH (10–12) was maintained by dropwise addition of NaOH (2 M) while stirring until precipitate appeared. The product was cooled to room temperature, filtered, and washed severally with C_2_H_5_OH and deionized water until the precipitate was free of alkali. The precipitate was dried at 105 °C in the oven for 5 h and transferred to the furnace at 550 °C for 18 h.

### 2.3. Synthesis of ZrFe_2_O_4_@ZIF-8

ZrFe_2_O_4_@ZIF-8 was prepared by sonicating a mixture of methanolic solutions (20.00 mL) of Zn(NO_3_)_2_·6H_2_O (0.293 g, 0.985 mmol) and C_4_H_6_N_2_ (0.809 g, 9.85 mmol) which contained ZrFe_2_O_4_ (50.00 mg). The mixture was sonicated (20 min) and further stirred (15 min) at room temperature and a speed of 120 rpm. The product was centrifuged (5500 rpm, 10 min) thrice, washing with ethanol. The ZrFe_2_O_4_@ZIF-8 obtained was dried overnight at room temperature. ZrFe_2_O_4_@ZIF-8 was activated at 100 °C for 3 h before its use for photocatalytic degradation of DOP and SMX.

### 2.4. Characterization of ZrFe_2_O_4_ and ZrFe_2_O_4_@ZIF-8 particles

The functional groups in ZrFe_2_O_4_ and ZrFe_2_O_4_@ZIF-8 were evaluated by taking spectra readings at 400–4500 cm^−1^ on Fourier-transformed infrared spectroscopy (FTIR, PerkinElmer, RXI 83303, USA). Their thermal stability was analyzed *via* thermogravimetric analysis (TGA) on TGA/DSC 2 Star^e^ system (DB V1300A-ICTA-Star^e^), and the diffraction pattern was recorded using X-ray diffractometer (2*θ*) read at 5–90° with filtered Cu Kβ radiation. The activity of ZrFe_2_O_4_ and ZrFe_2_O_4_@ZIF-8 in the UV-visible light region was recorded using a UV-visible spectrophotometer, while the surface morphology and elemental composition were determined using SEM (JEOL JSM-5510LV) equipped with energy-dispersive X-ray spectroscopy (EDS) (INCA mics EDX system). TEM images were taken on Talos F200X G2.

### 2.5. Photocatalytic degradation of DOP and SMX by ZrFe_2_O_4_ and ZrFe_2_O_4_@ZIF

The removal of DOP and SMX from the solution was achieved *via* photocatalytic degradation using ZrFe_2_O_4_ and ZrFe_2_O_4_@ZIF-8 under visible light with the help of a solar simulator (Xe, 150 W) possessing filter holder.^36^ Test solutions (50 mL) of DOP (5.00 mg L^−1^) and SMX (5.00 mg L^−1^) were contacted separately with ZrFe_2_O_4_ (0.1 g) or ZrFe_2_O_4_@ZIF-8 (0.1 g) in a beaker (100 mL) under visible light irradiation while stirring at 120 rpm for 180 min. The distance between the test solution and the solar simulator lamp was maintained at 20 cm. Samples of DOP or SMX from the degrading test solutions were withdrawn at an interval to evaluate the degradation capacity of ZrFe_2_O_4_ or ZrFe_2_O_4_@ZIF-8. The drawn samples were analyzed using a UV-visible spectrophotometer (PerkinElmer, Lambda). The photodegradation was established after taking UV-visible measurements at a predetermined wavelength: DOP (*λ*_max_ = 280 nm) and SMX (*λ*_max_ = 257 nm). Based on the better performance of ZrFe_2_O_4_@ZIF-8, further studies on process parameters, including the effect of weight (0.1 to 0.5 g), the concentration of DOP and SMX (1.00 to 5.00 mg L^−1^) and pH (2–10) on the photodegradation of DOP and SMX were only carried out using ZrFe_2_O_4_@ZIF-8. A dark experiment was conducted to check the impact of adsorption on the photodegradation process. The dark experiment included a concentration (DOP or SMX) of 5.00 mg L^−1^, a weight of 0.1 g (ZrFe_2_O_4_@ZIF-8) and a solution pH of 7.2 without light irradiation. All the experiments were conducted thrice, and values are presented as a mean of triplicate readings. The degradation efficiency was calculated as follows:1
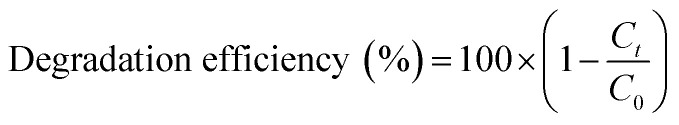
where *C*_0_ is the initial concentration of the test solutions of DOP or SMX and *C*_*t*_ is the concentration of the test solutions of DOP or SMX at time *t*. For the dark experiment, the adsorption capacity (*q*_e_) and the percentage removal (% removal) expressed towards DOP and SMX were calculated as follows:2
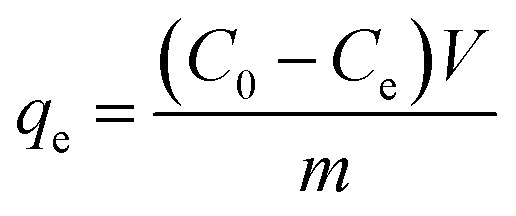
3
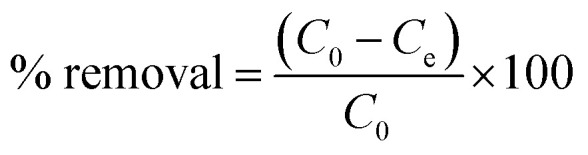
where *C*_0_ (mg L^−1^) represents the initial concentration of the test solution of DOP or SMX, *C*_e_ (mg L^−1^) is the test solution concentrations of DOP or SMX at equilibrium; *V* (in litre) represents the solution volume, the weight (g) of ZrFe_2_O_4_@ZIF-8 is defined as *m*, and the adsorption capacity is given as *q*_e_ (mg g^−1^).

### 2.6. Scavenging of reactive oxygen species

To understand the mechanism of action of ZrFe_2_O_4_@ZIF-8, the role of reactive oxygen species (ROS) during the degradation of DOP and SMX under visible light irradiation was investigated. The investigation estimated ammonium oxalate (AO) as a hole (h^+^) scavenger, isopropyl alcohol (IPA) as a scavenger of hydroxyl radical (OH·) and chloroform (CH) representing scavenger of superoxide ion radical (˙O_2_^−^). The role played by the scavengers during the degradation was determined by separately including each in the test solution at a concentration of 1 mM while conducting the photodegradation process. All the process conditions, such as test solution concentrations of DOP and SMX (5.00 mg L^−1^), a weight of ZrFe_2_O_4_@ZIF-8 (0.1 g), process time (180 min) and solution pH (7.2) for the photodegradation process were maintained.

### 2.7. Regeneration for reuse and stability of ZrFe_2_O_4_@ZIF-8

The regeneration of ZrFe_2_O_4_@ZIF-8 was determined *via* solvent desorption at the end of the photodegradation process. ZrFe_2_O_4_@ZIF-8 was filtered from the treated test solution at the end of the degradation process and washed with different solvent systems, including deionized water (H_2_O), HCl (0.1 M), methanol (MeOH) or a mixture of MeOH and 0.1 M HCl (1 : 3). The cleaned ZrFe_2_O_4_@ZIF-8 was oven dried at 105 °C for 5 h and reused for the photodegradation of DOP and SMX. Samples were withdrawn from the treated test solutions of DOP or SMX and analyzed with inductively coupled plasma optical emission spectroscopy (ICP-OES) to check whether ZrFe_2_O_4_@ZIF-8 leached into the treated test solutions of DOP and SMX during the photodegradation process. The stability of ZrFe_2_O_4_@ZIF-8 during the photodegradation of DOP and SMX was determined in ten (10) successive operational cycles by subjecting ZrFe_2_O_4_@ZIF-8 to XRD and FTIR analyses at the end of each cycle to check whether there was a change in structural pattern of ZrFe_2_O_4_@ZIF-8. Furthermore, ICP-OES analysis of the treated test solution was conducted at the end of each cycle.

## 3 Results and discussion

### 3.1. Synthesis and characterization of ZrFe_2_O_4_ and ZrFe_2_O_4_@ZIF-8 particles

**Fig. 1:**
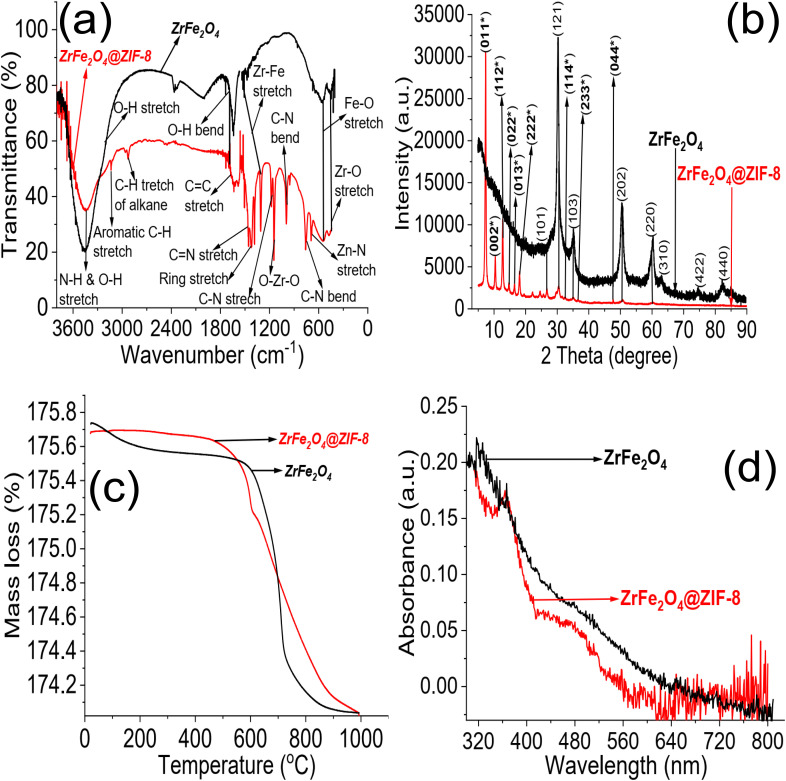
FTIR (a), XRD (b), TGA (c) and UV-visible spectra (d) of ZrFe_2_O_4_ and ZrFe_2_O_4_@ZIF-8.

Different techniques characterized the synthesis of ZrFe_2_O_4_ and ZrFe_2_O_4_@ZIF-8. The FTIR spectra of ZrFe_2_O_4_ and ZrFe_2_O_4_@ZIF-8 are presented in Fig. 1a. The peak at 3431 cm^−1^ suggests the O–H stretch due to adsorbed water molecules in ZrFe_2_O_4_ and ZrFe_2_O_4_@ZIF-8 however, the signal is broader in ZrFe_2_O_4_@ZIF-8 which means an overlap from N–H stretch of imidazole structure of ZrFe_2_O_4_@ZIF-8. The peak at 3243 cm^−1^ appeared only in ZrFe_2_O_4_@ZIF-8, which may be attributed to the C–H stretch of an aromatic ring. Furthermore, the peak at 2935 cm^−1^ occurred in the spectra of ZrFe_2_O_4_ and ZrFe_2_O_4_@ZIF-8, which may be assigned to the C–H stretch of alkane. The signal at 1635 cm^−1^ in both ZrFe_2_O_4_ and ZrFe_2_O_4_@ZIF-8 is attributed to the O–H bend of adsorbed water molecules, although this signal is weak in ZrFe_2_O_4_@ZIF-8. The bands at 1641 and 1558 cm^−1^ in ZrFe_2_O_4_@ZIF-8 were attributed to C=C and C=N stretching, respectively, while the imidazole ring stretches in ZrFe_2_O_4_@ZIF-8 were seen at 1438 cm^−1^. Corresponding signals at 1393 and 1398 cm^−1^ in ZrFe_2_O_4_ and ZrFe_2_O_4_@ZIF-8 were assigned to Fe–Zr stretch, respectively, while the signal at 1198 cm^−1^ was due to C–N stretch in ZrFe_2_O_4_@ZIF-8. The O–Zr–O stretch appeared at 1191 cm^−1^ in ZrFe_2_O_4_ and ZrFe_2_O_4_@ZIF-8, while the C–N bend signal was only seen at 1006 cm^−1^ in ZrFe_2_O_4_@ZIF-8. The peak at 651 cm^−1^ was attributed to the Zn–N stretch of the imidazole structure in ZrFe_2_O_4_@ZIF-8, while signals at 621 and 562 cm^−1^ in ZrFe_2_O_4_ and ZrFe_2_O_4_@ZIF-8 were due to Fe–O and Zr–O vibrations, respectively.

The XRD results for ZrFe_2_O_4_ and ZrFe_2_O_4_@ZIF-8 revealed signals corresponding to (011), (002), (112), (022), (013), (222), (101), (121), (114), (103), (233), (004), (202), (220), (310), (422) and (440). The diffraction patterns from the signals confirmed the synthesis of ZrFe_2_O_4_ and ZrFe_2_O_4_@ZIF-8. Signals that were not seen in ZrFe_2_O_4_ are asterisked (Fig. 1b), suggesting that they only appeared in ZrFe_2_O_4_@ZIF-8 which confirmed the presence of imidazole structure (from ZIF-8) in ZrFe_2_O_4_@ZIF-8.^40,41^ The crystallite sizes of ZrFe_2_O_4_ and ZrFe_2_O_4_@ZIF-8 were determined from their broadening line of reflections according to Debye–Scherrer's formula:^42^4
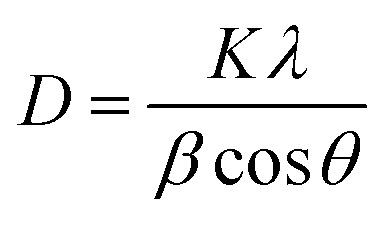


The average crystallite sizes of ZrFe_2_O_4_ and ZrFe_2_O_4_@ZIF-8 are denoted as *D*, *K* is a constant (0.89) while *λ* (1.5406 Å) is the X-ray wavelength. The entire width of the diffraction line and Bragg's angle taken at the peak are represented as *β* and *θ*, respectively.^43^ The crystallite size of ZrFe_2_O_4_@ZIF-8 (26.10 nm) is larger than that of ZrFe_2_O_4_ (21.23 nm), which may be due to a larger molecular size of ZrFe_2_O_4_@ZIF-8 from the incorporation of ZIF-8 in its structure. This may have caused an extension in the bulk crystallite size, which is described by the diffusion properties exhibited by ZrFe_2_O_4_ and ZrFe_2_O_4_@ZIF-8:^44,45^

5 *τ* = *r*^2^π^2^*D*

The average diffusion time to the surface of ZrFe_2_O_4_ and ZrFe_2_O_4_@ZIF-8 is *τ* while *D* is the diffusion coefficient. From the expression, *τ* gets longer when *D* becomes large; this possibility puts the particles at risk of aggregation or recombination when functioning as a catalyst. The capacity of the particles to act as a catalyst becomes hampered when aggregation or recombination occurs among the particles.^45,46^ Therefore, for optimum catalytic performance, the crystallite size should be small.^47^ Interestingly, the crystallite size exhibited by ZrFe_2_O_4_ and ZrFe_2_O_4_@ZIF-8 is smaller than the range (37 to 45 nm) reported for spinel ferrites,^47^ suggesting ZrFe_2_O_4_ and ZrFe_2_O_4_@ZIF-8 as potential photocatalysts.

**Fig. 2:**
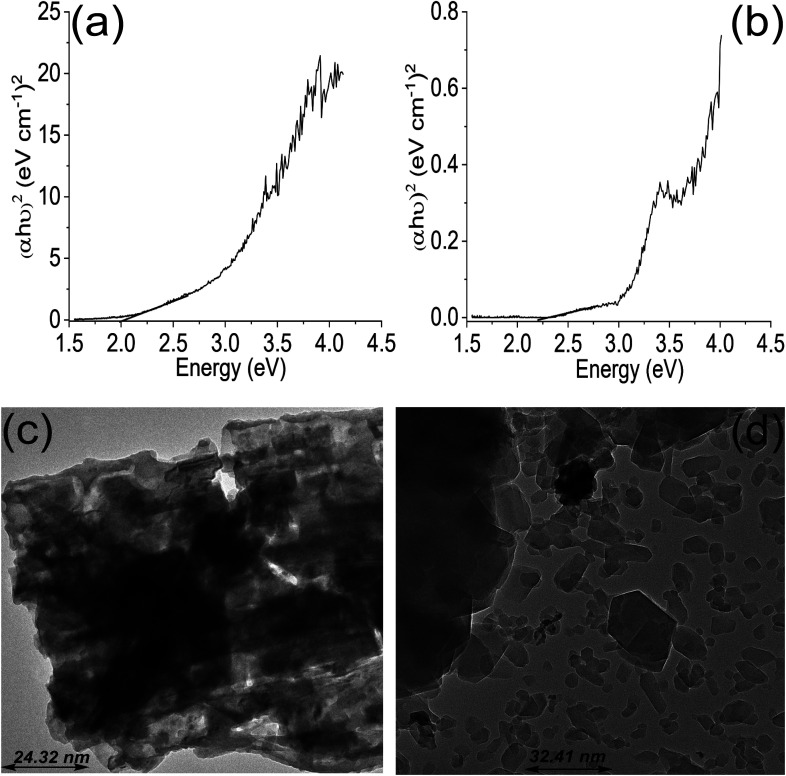
Tauc plot for ZrFe_2_O_4_ (a), Tauc plot for ZrFe_2_O_4_@ZIF-8 (b), TEM of ZrFe_2_O_4_ (c) and TEM of ZrFe_2_O_4_@ZIF-8 (d).

The TGA results showed distinct phase losses in ZrFe_2_O_4_ and ZrFe_2_O_4_@ZIF-8 (Fig. 1c). The mass loss at 60 to160 °C suggests loss of adsorbed water molecules (peaks at 3243 and 1635 cm^−1^ in the FTIR results) and volatile molecule adsorbed on the surfaces of ZrFe_2_O_4_ and ZrFe_2_O_4_@ZIF-8. There is a mass loss from 160 to 630 °C in ZrFe_2_O_4_, which may be due to the formation of metal oxides and dehydration of the OH group in its spinel structure involving inter and intramolecular transfer reactions.^48,49^ The mass loss from 630 to 710 °C (ZrFe_2_O_4_) and 510 to 900 °C (ZrFe_2_O_4_@ZIF-8) may be attributed to phase change and decomposition of ZIF-8 structure, respectively. Mass loss above 900 °C in ZrFe_2_O_4_@ZIF-8 may be attributed to structural collapse and carbonization,^50^ while mass loss above 710 °C in ZrFe_2_O_4_ may be due to phase change. ZrFe_2_O_4_ and ZrFe_2_O_4_@ZIF-8 exhibited activity in the visible light region of the spectrum, as shown in Fig. 1d, indicating that they may both exhibit photocatalytic activity within this region of the light spectrum. This indication led us to probe the possibility of using ZrFe_2_O_4_ and ZrFe_2_O_4_@ZIF-8 for the photodegradation of DOP and SMX. The band gaps were calculated from the Tauc plot for ZrFe_2_O_4_ (Fig. 2a) and ZrFe_2_O_4_@ZIF-8 (Fig. 2b) as follows:

6 (*αhv*)^2^ = *A*(*hv* − *E*_g_)

Where *hv* represents the frequency of light from the solar irradiator, the proportionality constant is defined as *A*, the bandgap is denoted as *E*_g_ and *α* represent the absorption coefficient. The result indicated that the bandgap of ZrFe_2_O_4_@ZIF-8 (2.10 eV) is higher than that of ZrFe_2_O_4_ (2.05 eV), which may be due to the inclusion of ZIF-8 in the structure of ZrFe_2_O_4_ to produce ZrFe_2_O_4_@ZIF-8. Fortunately, these values obtained for ZrFe_2_O_4_@ZIF-8 (2.10 eV) and ZrFe_2_O_4_ (2.05 eV) are within the value range suitable for visible light active photocatalysts.^51,52^

The average particle size of ZrFe_2_O_4_ and ZrFe_2_O_4_@ZIF-8 were found to be 24.32 nm and 32.41 nm, respectively, from the TEM images. The average particle size increased in ZrFe_2_O_4_@ZIF-8, which may be due to the imidazole structure of the ZIF-8 that increases the molecular weight. The particles exhibit irregular sizes and shapes.

**Fig. 3:**
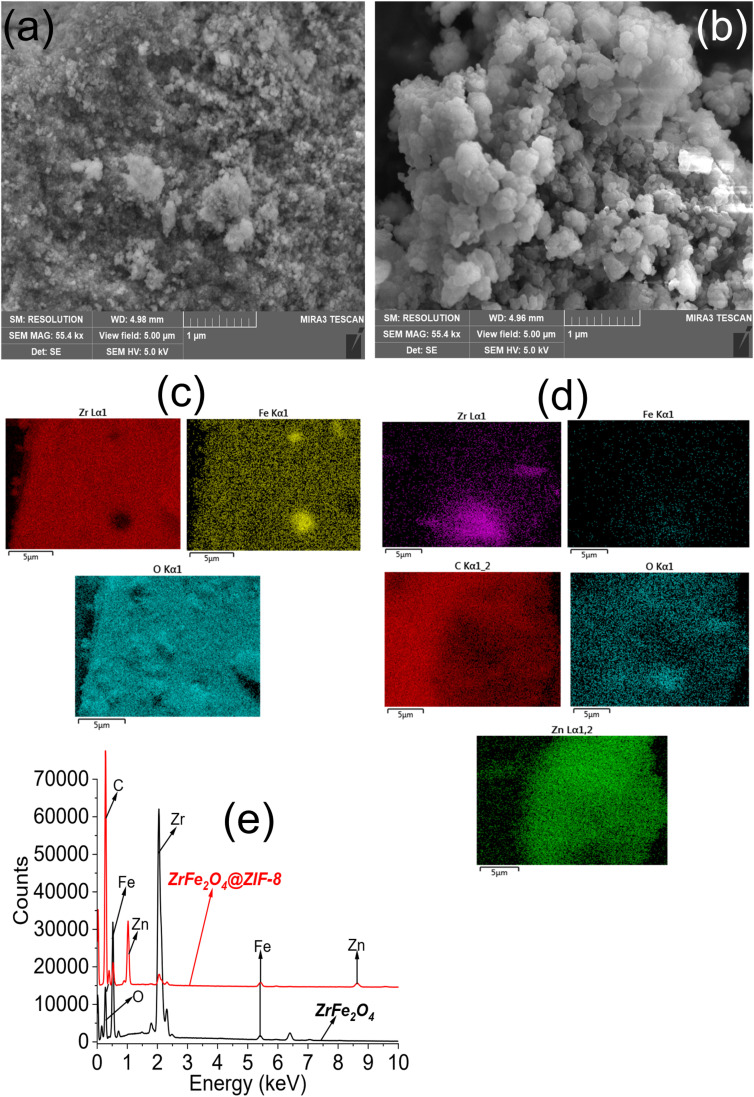
SEM of ZrFe_2_O_4_ (a) SEM of ZrFe_2_O_4_@ZIF-8 (b), elemental mapping of ZrFe_2_O_4_ (c), elemental mapping of ZrFe_2_O_4_@ZIF-8 (d) and EDS of ZrFe_2_O_4_ and ZrFe_2_O_4_@ZIF-8 (e).

The SEM images showed the surfaces of ZrFe_2_O_4_ (Fig. 3a) and ZrFe_2_O_4_@ZIF-8 (Fig. 3b) to be heterogeneous. The surface of ZrFe_2_O_4_ revealed stacked particles, whereas the pores appeared more open in ZrFe_2_O_4_@ZIF-8 with particles that are less stacked together when compared with ZrFe_2_O_4_. Nonetheless, the particles appeared to be agglomerated in both ZrFe_2_O_4_ and ZrFe_2_O_4_@ZIF-8. The elemental surface mappings are shown in Fig. 3c (ZrFe_2_O_4_) and Fig. 3d (ZrFe_2_O_4_@ZIF-8), which describe the type of elements present in their particles. Furthermore, the EDS results (Fig. 3e) confirmed the elemental composition of ZrFe_2_O_4_ to be zirconium (Zr), iron (Fe) and oxygen (O). Similar elements were established in ZrFe_2_O_4_@ZIF-8 with the inclusion of carbon (C) and zinc (Zn) emanating from the imidazole structure (ZIF-8).

### 3.2. Photodegradation of DOP and SMX

**Fig. 4:**
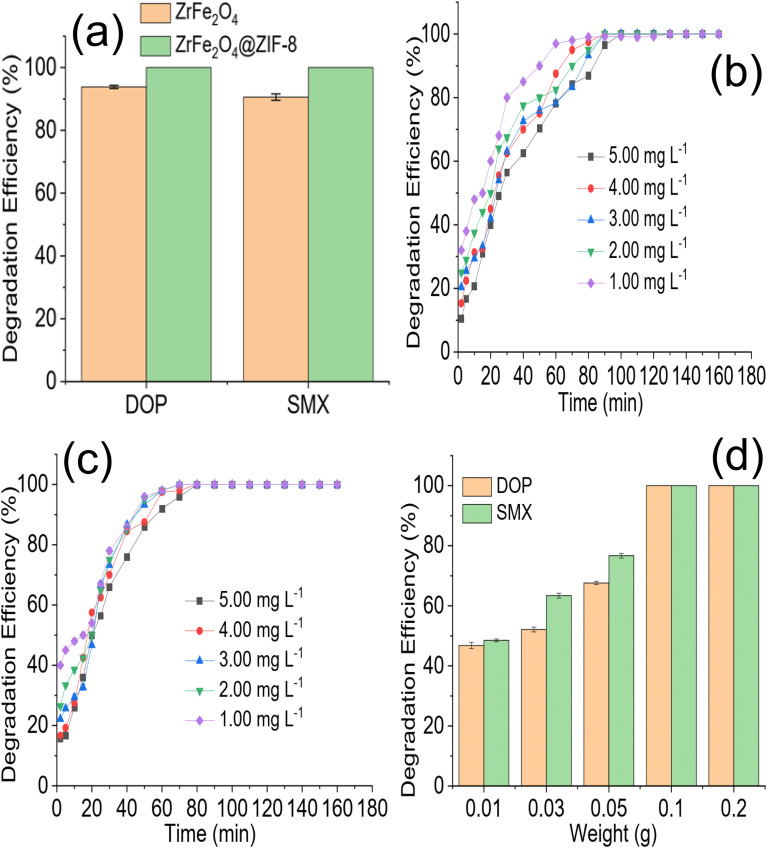
Comparison of the preliminary degradation efficiency expressed by ZrFe_2_O_4_ and ZrFe_2_O_4_@ZIF-8 towards DOP and SMX (a), time dependent degradation of DOP in the presence of ZrFe_2_O_4_@ZIF-8 at different concentration (b), time dependent degradation of SMX in the presence of ZrFe_2_O_4_@ZIF-8 at different concentration (c) and effect of ZrFe_2_O_4_@ZIF-8 weight on the degradation of DOP and SMX (d).

The preliminary performance of ZrFe_2_O_4_ and ZrFe_2_O_4_@ZIF-8 (Fig. 4a) for the degradation of DOP and SMX revealed the degradation capacity of ZrFe_2_O_4_@ZIF-8 to be higher than that of ZrFe_2_O_4_. The degradation capacity expressed by ZrFe_2_O_4_ towards DOP was 93.85 ± 0.50% and 90.60 ± 1.00% towards SMX. On the other hand, ZrFe_2_O_4_@ZIF-8 expressed a complete (100%) degradation of DOP and SMX in the test solutions. Therefore, further studies for the degradation of DOP and SMX were conducted using ZrFe_2_O_4_@ZIF-8. The time-dependent degradation of DOP and SMX by ZrFe_2_O_4_@ZIF-8 are presented in Fig. 4b and c, respectively. In both DOP and SMX, the degradation efficiency expressed by ZrFe_2_O_4_@ZIF-8 increased with time. The initial degradation efficiency expressed by ZrFe_2_O_4_@ZIF-8 towards DOP and SMX is higher at low concentration (1.00 mg L^−1^) than at high concentration (5.00 mg L^−1^). The observation may be because at low concentrations, smaller amounts of DOP and SMX species are available in solution for ZrFe_2_O_4_@ZIF-8 to degrade, and as concentration increased from 1.00 to 5.00 mg L^−1^, the quantities of DOP and SMX species in solution increased requiring more activities of ZrFe_2_O_4_@ZIF-8 to ensure degradation.

The effect of ZrFe_2_O_4_@ZIF-8 weight on the degradation of DOP and SMX is shown in Fig. 4d. The degradation efficiency expressed by ZrFe_2_O_4_@ZIF-8 towards DOP and SMX increased with an increase in weight of ZrFe_2_O_4_@ZIF-8. This may be due to a rise in the surface area of ZrFe_2_O_4_@ZIF-8 as its weight increased from 0.01 to 0.2 g. Increasing the weight must have increased the number of active sites available for the degradation process, thereby increasing the efficiency of ZrFe_2_O_4_@ZIF-8 as weight increased. Similar observations as been previously reported.^36^ Any attempt to increase the weight of ZrFe_2_O_4_@ZIF-8 beyond 0.2 g led to a decrease in the activity of ZrFe_2_O_4_@ZIF-8, which may be attributed to the fact that as the weight of ZrFe_2_O_4_@ZIF-8 increased beyond 0.2 g, the penetration of irradiated light rays reduced. The reduction in light penetration due to the bulkiness of ZrFe_2_O_4_@ZIF-8 as weight increased may have prevented the excitation of ZrFe_2_O_4_@ZIF-8 because of the shielding effect resulting from the excessive scattering of the photons at the surface of ZrFe_2_O_4_@ZIF-8.^53^

**Fig. 5:**
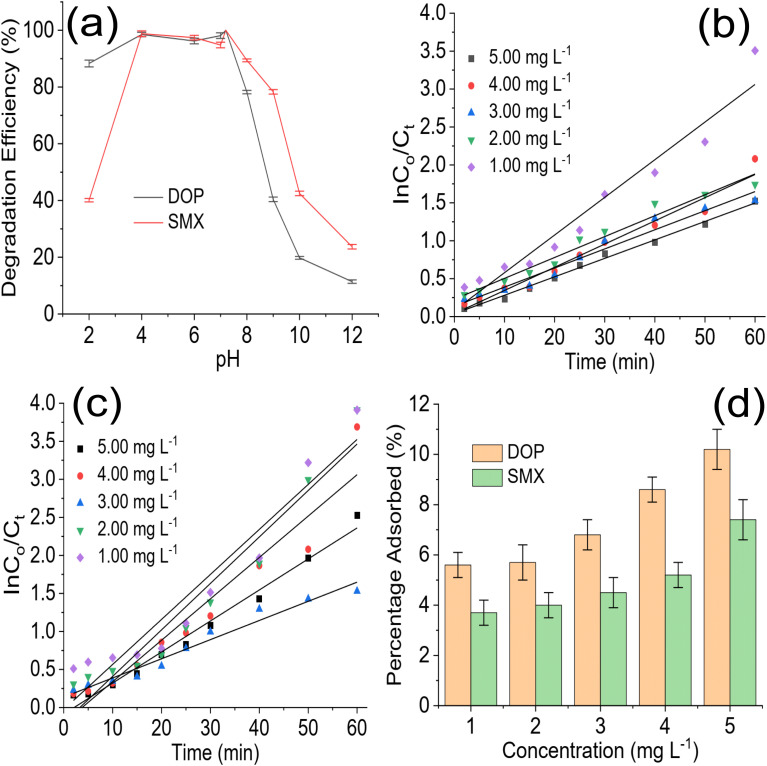
Effect of solution pH on the degradation of DOP and SMX by ZrFe_2_O_4_@ZIF-8 (a), plot of ln *C*_0_/*C*_*t*_*versus* irradiation time for the degradation of DOP (b) and SMX (c) at different solution concentrations in the presence of ZrFe_2_O_4_@ZIF-8 and percentage adsorbed during degradation of DOP and SMX by ZrFe_2_O_4_@ZIF-8 in the dark experiment (d).

The role of pH in photodegradation should be investigated because acidity and alkalinity play an essential role in catalyst behaviour in a reaction medium. The test solution pH was varied from 2 to 12 (Fig. 5a) to understand the role of pH in the degradation of DOP and SMX by ZrFe_2_O_4_@ZIF-8. As the pH of the test solution was increased from 2 to 7, the performance of ZrFe_2_O_4_@ZIF-8 was enhanced, and more DOP and SMX were removed from the solution. Unfortunately, the performance of ZrFe_2_O_4_@ZIF-8 decreased as the pH increased after pH 7.2. Therefore, the best pH for the degradation of DOP and SMX by ZrFe_2_O_4_@ZIF-8 is 7.2. As pH increased towards 7.2, more ROS were available in the test solution for the degradation process. Degradation data were fitted for the pseudo-first-order kinetic model to understand the rate of the degradation process as:7
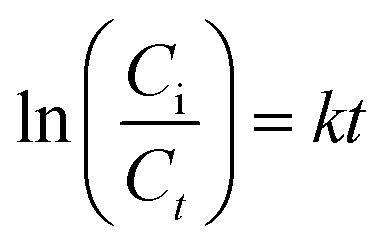
where *C*_i_ and *C*_*t*_ are the initial concentrations of DOP and SMX and concentrations of DOP and SMX at a specific time “*t*”, respectively, *K* denotes the pseudo-first-order rate constant generated from the plot of ln *C*_i_/*C*_*t*_*versus* time, and *t* is the irradiation time. The photodegradation rate for ZrFe_2_O_4_@ZIF-8 towards DOP and SMX was determined from the plot of ln *C*_i_/*C*_*t*_*versus* visible light irradiation time at the different concentrations of DOP and SMX (Fig. 5b and c).

The photodegradation rate constant expressed for the degradation of DOP increased with a decrease in test solution concentration (5.00 mg L^−1^ = 0.0243 min^−1^, 4.00 mg L^−1^ = 0.0306 min^−1^, 3.00 mg L^−1^ = 0.0352 min^−1^, 2.00 mg L^−1^ = 0.0375 min^−1^ and 1.00 mg L^−1^ = 0.0497 min^−1^). It was also observed in the initial degradation efficiency expressed by ZrFe_2_O_4_@ZIF-8 towards DOP (Fig. 4b). The degradation efficiency was highest for the low concentrations at initial treatment time; furthermore, it took a shorter time for ZrFe_2_O_4_@ZIF-8 to completely degrade DOP at the least concentration (1.00 mg L^−1^) than for the higher concentrations. A similar result was obtained for the degradation of SMX (5.00 mg L^−1^ = 0.0592 min^−1^, 4.00 mg L^−1^ = 0.0611 min^−1^, 3.00 mg L^−1^ = 0.0619 min^−1^, 2.00 mg L^−1^ = 0.0644 min^−1^ and 1.00 mg L^−1^ = 0.0645 min^−1^). It may be concluded that the rate of photocatalytic degradation of DOP and SMX by ZrFe_2_O_4_@ZIF-8 is fastest at low concentrations of DOP and SMX, which may be due to the low amounts of DOP and SMX species in solution at such low concentrations. A dark experiment was conducted to investigate the effect of adsorption on the photodegradation process. Interestingly, ZrFe_2_O_4_@ZIF-8 demonstrated more affinity for DOP than SMX (Fig. 5d). During the dark experiment, the adsorption of DOP by ZrFe_2_O_4_@ZIF-8 increased with an increase in concentration (1.00 to 5.00 mg L^−1^) from 5.60 ± 0.50 to 10.20 ± 0.80%, similarly, in the case of SMX, the adsorption of SMX increased from 3.70 ± 0.50 to 7.40 ± 0.80% with an increase in concentration (1.00 to 5.00 mg L^−1^). The adsorption capacity expressed by ZrFe_2_O_4_@ZIF-8 towards DOP and SMX is 0.51 and 0.37 mg g^−1^, respectively. This revealed that adsorption and photocatalysis took place simultaneously while removing DOP and SMX from the solution. In both degradations of DOP and SMX, the percentage removal of DOP and SMX *via* the adsorption process is less than 15% of the total performance of ZrFe_2_O_4_@ZIF-8.

### 3.3. Proposed mechanism for the photodegradation of DOP and SMX

**Fig. 6:**
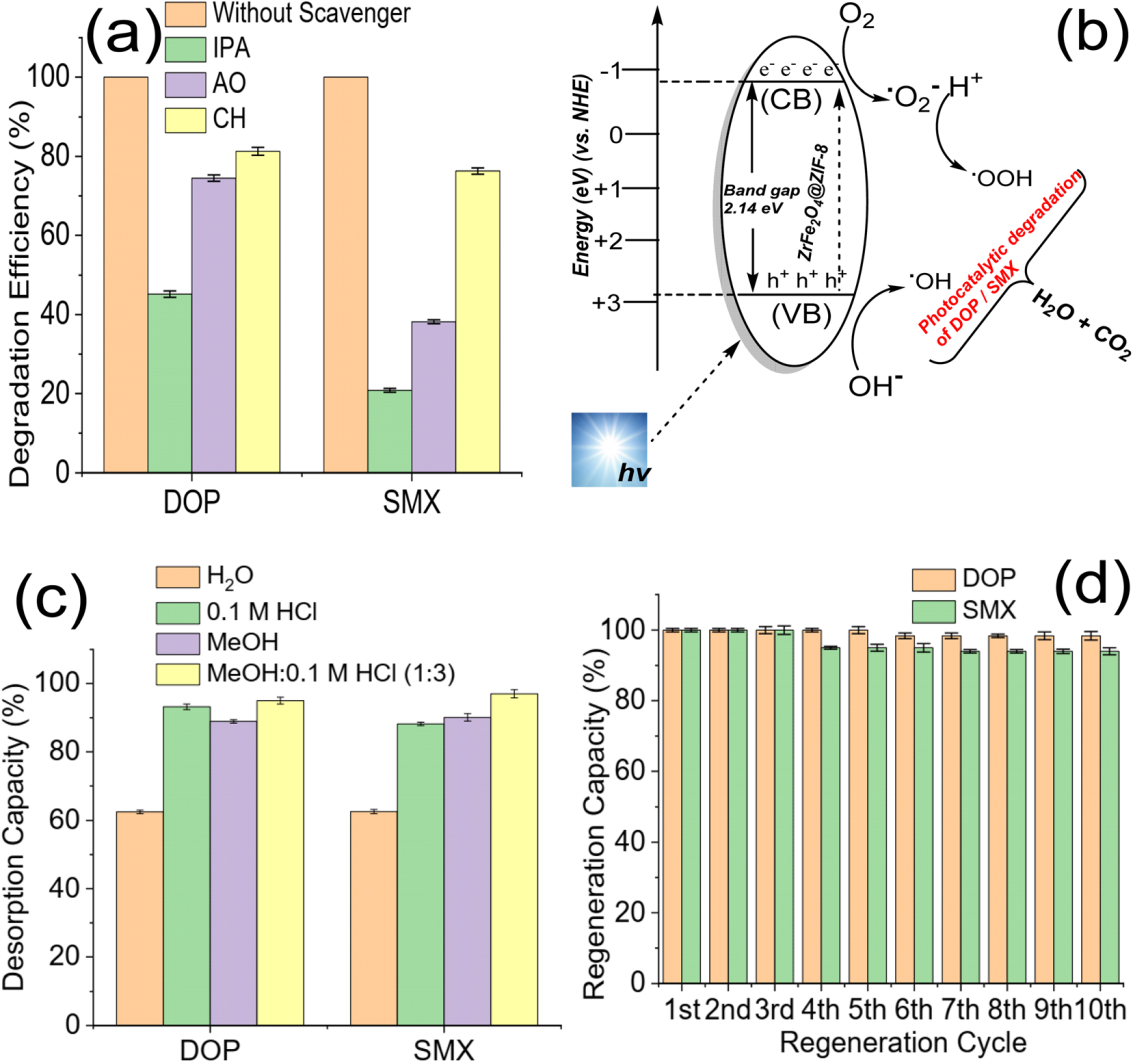
Degradation efficiency of ZrFe_2_O_4_@ZIF-8 towards DOP and SMX with and without ROS scavengers (a), proposed mechanism for the photodegradation of DOP and SMX (b), desorption efficiency of ZrFe_2_O_4_@ZIF-8 after washing with different solvent systems (c) and regeneration capacity of ZrFe_2_O_4_@ZIF-8 expressed towards DOP and SMX at different treatment cycle (d).

The role of ROS may explain the degradation of DOP and SMX by ZrFe_2_O_4_@ZIF-8. Previous studies have attributed the photocatalytic degradation of organic molecules to the involvement of ROS.^53^ Therefore, to understand the mechanism of action of ZrFe_2_O_4_@ZIF-8 for the degradation of DOP and SMX, the degradation process was separately carried out in the presence of IPA (as a OH· scavenger), AO (as a h^+^ scavenger) and CH (as a ˙O_2_^−^ scavenger) as described.^36,54^ The performance of ZrFe_2_O_4_@ZIF-8 in the presence and absence of AO, IPA and CH were compared, as shown in Fig. 6a.

The performance of ZrFe_2_O_4_@ZIF-8 was least in the presence of IPA and highest in the presence of CH. This observation was found in the degradation of DOP and SMX, which suggests that OH· played a significant role in the degradation of DOP and SMX. The scavenging of the OH· by IPA led to a substantial decrease in the performance of ZrFe_2_O_4_@ZIF-8 as a photocatalyst for the degradation of DOP and SMX. On the contrary, degradation efficiency was highest for CH among the ROS scavengers studied, which suggests that ˙O_2_^−^ played the least role in the degradation of DOP and SMX. When CH was added to the test solution, the degradation efficiency exhibited by ZrFe_2_O_4_@ZIF-8 was 81.30 ± 1.0 and 76.30 ± 0.80% towards DOP and SMX, respectively whereas, when IPA was added to the test solution, these values were 45.20 ± 0.80 and 20.80 ± 0.50% towards DOP and SMX. Therefore, the degradation efficiency of ZrFe_2_O_4_@ZIF-8 (in the presence of ROS scavenger) is inversely proportional to the magnitude of the role played by the ROS scavenged.

In the present study, scavenging the OH· by IPA gave the least performance suggesting that it played a significant role in degrading DOP and SMX. The mechanism for the degradation of DOP and SMX by ZrFe_2_O_4_@ZIF-8 is *via* ˙OH, h^+^, and ˙O_2_^−^ generation (Fig. 6b) in the test solution when visible light is shone on the degrading system. During the process, ZrFe_2_O_4_@ZIF-8 absorbs visible light to generate h^+^ from the valence band (VB) and e^−^ from the conduction band (CB). H^+^ and ˙OH are produced in the test solution from the reaction of h^+^ with water molecules, and subsequently, ˙O_2_^−^ is produced from O_2_ as a result of the reaction of e^−^. The generated ROS initiates and propagates the degradation process. Unfortunately, h^+^ and e^−^ often recombine, leading to loss of ROS generation, which is disadvantageous to the degradation process. The recombination of h^+^ and e^−^ was inhibited with the presence of ZIF-8 in the structure of ZrFe_2_O_4_@ZIF-8. The ZIF-8 serves as a carbon source to slow down the recombination process. During this process, the carbon source (ZIF-8) is an acceptor for trapping the generated h^+^ and e^−^ to inhibit their migration for combination. Therefore, when they are trapped, they become fixed at a point, making them less mobile and preventing interaction between the h^+^ and e^−^, as previously demonstrated in a study where a carbon dot was used as a source of carbon.^53,55–57^ This approach helped prevent the premature recombination of h^+^ and e^−^.

### 3.4. Regeneration for reuse and stability of ZrFe_2_O_4_@ZIF-8

**Fig. 7:**
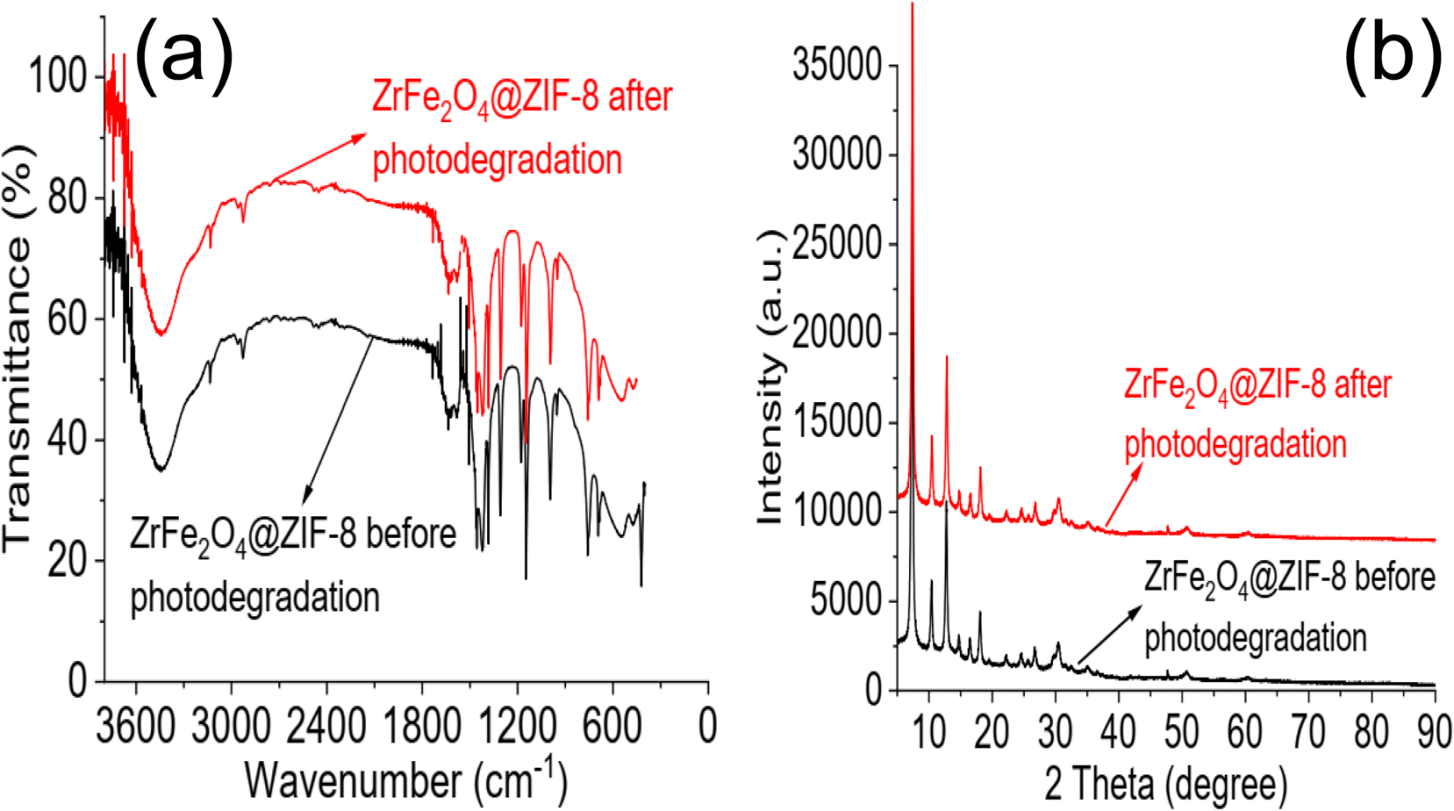
FTIR of ZrFe_2_O_4_@ZIF-8 before photodegradation and at 10^th^ cycle of photodegradation (a) and XRD of ZrFe_2_O_4_@ZIF-8 before photodegradation and at 10^th^ cycle of photodegradation (b).

The regeneration of ZrFe_2_O_4_@ZIF-8 for reuse is essential as it helps determine its economic viability and affordability. The regeneration of ZrFe_2_O_4_@ZIF-8 for reuse was studied *via* desorption with solvent. Spent ZrFe_2_O_4_@ZIF-8 was subjected to desorption using solvents (H_2_O), 0.1 M HCl, MeOH and a mixture of MeOH and 0.1 M HCl (1 : 3). The solvents were selected based on the solubility of DOP and SMX. As shown in Fig. 6c, the best solvent for the regeneration of ZrFe_2_O_4_@ZIF-8 is a mixture of MeOH : 0.1 M HCl (1 : 3). The desorption capacity was 95.00 ± 1.00% for DOP and 97 ± 1.20% for SMX. The stability was conducted in 10 regeneration cycles using MeOH : 0.1 M HCl (1 : 3) as solvent (Fig. 6d). ZrFe_2_O_4_@ZIF-8 showed good stability for reuse with a regeneration capacity of 98.40 ± 1.20% for DOP and 94 ± 1.00% for SMX even at the 10^th^ regeneration cycle. The regeneration capacity was 100% up until the 5^th^ regeneration cycle for DOP before it dropped to 98.40 ± 1.20% and remained steady again until the 10^th^ cycle. Similarly, the regeneration capacity was 100% for SMX until the 4^th^ regeneration cycle before dropping to 94.00 ± 1.00% and remaining consistent until the 10^th^ cycle. The stability was further investigated by subjecting ZrFe_2_O_4_@ZIF-8 to FTIR (Fig. 7a) and XRD (Fig. 7b) analysis at the end of the 10^th^ cycle to check whether there were any changes in the structure of ZrFe_2_O_4_@ZIF-8. Comparing the spectra before and after photocatalytic degradation of DOP and SMX showed no changes in the structural pattern of ZrFe_2_O_4_@ZIF-8, confirming its stability. Furthermore, Fe, Zn and Zr were not detected from the results of the ICP-OES analysis of the treated water sample.

The performance of ZrFe_2_O_4_@ZIF-8 was compared with previously published works in the literature. Unfortunately, studies on the degradation of DOP are rare; however, there are studies on SMX for comparison, as shown in Table 1. A recent study demonstrated an immobilized biomass reactor's enhanced performance of about 93% towards SMX.^58^ However, the reactor's process conditions and time of operation make it expensive and non-sustainable in developing countries. Permanganate combined with bisulfite has been shown to have a rapid removal capacity for SMX;^59^ however, ZrFe_2_O_4_@ZIF-8 exhibited a complete removal of SMX. *Sphingobacterium mizutaii* LLE5 has demonstrated capacity for removing SMX under optimal degradation conditions with a capacity of 93.87%,^60^ which is lower than the efficiency (100%) demonstrated by ZrFe_2_O_4_@ZIF-8. A recent study reported bowl-like FeCuS@Cu_2_S@Fe^0^ with excellent complete removal of SMX within 5 min of operation. However, the synthetic route and stability exhibited by ZrFe_2_O_4_@ZIF-8 is an additional advantage over FeCuS@Cu_2_S@Fe^0^. ZrFe_2_O_4_@ZIF-8 compared favourably with previously reported photocatalysts. The degradation efficiency shown by ZrFe_2_O_4_@ZIF-8 is higher than values reported for AgNbO_3_,^33^ ZnO (ref. 61) and CuO_*x*_–BiVO_4_ (ref. 62) for the degradation of SMX. The stability of ZrFe_2_O_4_@ZIF-8 is better than most reported photocatalysts; ZrFe_2_O_4_@ZIF-8 expressed a capacity of 94.00% at the 10^th^ cycle of treatment which is higher than that of Co–CHNTs^63^ and Er^3+^/Tb^3+^@BiOBr-gC_3_N_5_.^64^ The high stability and reusability of ZrFe_2_O_4_@ZIF-8 for the degradation of DOP and SMX make it promising for treating contaminated water systems.

**Table 1:** Comparison of the photodegradation of DOP and SMX by ZrFe_2_O_4_@ZIF-8 with other photocatalysts in literature^a^

Material	Antibiotic	DE (%)	LIS	AC (g L^−1^)	Conc. (mg L^−1^)	Stability (%)	Reference
ZnO@g-C_3_N_4_	SMX	90.40	UVC lamp	0.65	30.00	—	65
Biochar-supported TiO_2_	SMX	91.00	UVC lamp	5.00	10.00	—	66
ZnO	SMX	80.00	UVC lamp	1.50	10.00	—	61
g-C_3_N_4_@ZnO	SMX	94.20	Xe lamp	0.40	10.00	—	34
CuO_*x*_–BiVO_4_	SMX	40.00	Xe ozone-free lamp	0.50	0.50	—	62
AgNbO_3_	SMX	98.00	Fluorescent lamps	0.50	10.00	82.00 (3^rd^ cycle)	33
FPTC	SMX	98.50	Sunlight	1.00	30.00	—	8
Ag–P@UCN	SMX	99.00	8 W visible lamps	1.00	5.00	<10.00 (6^th^ cycle)	67
Ag_2_S/Bi_2_S_3_/g-C_3_N_4_	SMX	97.40	Xe lamp	0.025	20.00	97.40 (5^th^ cycle)	68
Ag_3_PO_4_/g-C_3_N_4_/BiVO_4_	SMX	93.60	250 W Xe lamp	0.005	20.00	>90.00 (4^th^ cycle)	69
AgI/MoO_3_	SMX	97.60	300 W Xe lamp	0.005	5.00	—	70
PTT	SMX	72.74	UV-LED	1.00	50.00	—	71
ZnO/Fe_2_O_3_	SMX	95.20	Xe lamp	0.30	10.00	—	72
Co–CuS@TiO_2_	SMX	100.00	Xe lamp	0.25	5.00	>90.00 (5^th^ cycle)	73
Co–CHNTs	SMX	97.50	Visible light	0.20	10.00	85.81 (4^th^ cycle)	63
Er^3+^/Tb^3+^@BiOBr-gC_3_N_5_	SMX	94.20	Visible light	0.075	10.00	83.60 (3^rd^ cycle)	64
ZrFe_2_O_4_@ZIF-8	DOP	100.00	150 W Xe light	0.02	5.00	98.40 (10^th^ cycle)	This study
	SMX	100.00		0.02	5.00	94.00 (10^th^ cycle)	

a — = not reported, DE = degradation efficiency, LIS = light illumination source, AC = amount of catalyst, Conc. = concentration, FPTC = F–Pd co-doped TiO_2_ nanocomposites, Ag–P@UCN = Ag-decorated phosphorus doped graphitic carbon nitride, PTT = porous titanium–titanium dioxide, DOP = dopamine, SMX = sulfamethoxazole.

## 4 Conclusion

The presence of pharmaceutical wastes such as DOP and SMX in water is an emerging global water challenge requiring urgent attention. In response, ZrFe_2_O_4_ and ZrFe_2_O_4_@ZIF-8 were synthesized and used to remove DOP and SMX in contaminated water. ZrFe_2_O_4_ and ZrFe_2_O_4_@ZIF-8 exhibited a crystallite size of 21.23 and 26.10 nm, respectively. The SEM images of ZrFe_2_O_4_ and ZrFe_2_O_4_@ZIF-8 revealed irregularly sized and shaped heterogeneous particles. The EDS results confirmed the elemental composition of ZrFe_2_O_4_ and ZrFe_2_O_4_@ZIF-8. The preliminary evaluation of ZrFe_2_O_4_ and ZrFe_2_O_4_@ZIF-8 for the degradation of DOP and SMX showed that the capacity of ZrFe_2_O_4_ is lower than that of ZrFe_2_O_4_@ZIF-8. The degradation capacity expressed by ZrFe_2_O_4_ towards DOP was 93.85 ± 0.50% and 90.60 ± 1.00% towards SMX. On the other hand, ZrFe_2_O_4_@ZIF-8 expressed a complete (100%) degradation of DOP and SMX in the test solutions. The study showed that the degradation process involved both adsorption and photocatalytic degradation simultaneously. ZrFe_2_O_4_@ZIF-8 demonstrated high stability with a consistent regeneration capacity of 98.40% for DOP and 94.00% for SMX at the 10^th^ cycle of treatment in a process described by pseudo-first-order kinetic. The study revealed ZrFe_2_O_4_@ZIF-8 as a promising photocatalyst for treating DOP and SMX-contaminated water.

## Author contributions

Adewale Adewuyi: conceptualization, project design and execution, formal analysis, investigation, writing and review, validation, and editing, Olaoluwa A. Ogunkunle: validation, analysis, Rotimi A. Oderinde: conceptualization, editing, analysis, validation, and review.

## Conflicts of interest

The authors declare that they have no known competing financial interests or personal relationships that could have appeared to influence the work reported in this paper.

## Supplementary Material

RA-013-D3RA01055D-s001
