# Supplementary material for: Zirconium ferrite incorporated zeolitic imidazolate framework-8: a suitable photocatalyst for degradation of dopamine and sulfamethoxazole in aqueous solution
Source: RSC Adv. 2023 Mar 23;13(14):9563–75. doi: 10.1039/d3ra01055d (PMC10035307; doi:10.1039/d3ra01055d)
Supplement: RA-013-D3RA01055D-s001 [file RA-013-D3RA01055D-s001.pdf]

Electronic Supplementary Material (ESI) for RSC Advances.
This journal is © The Royal Society of Chemistry 2023

Figures

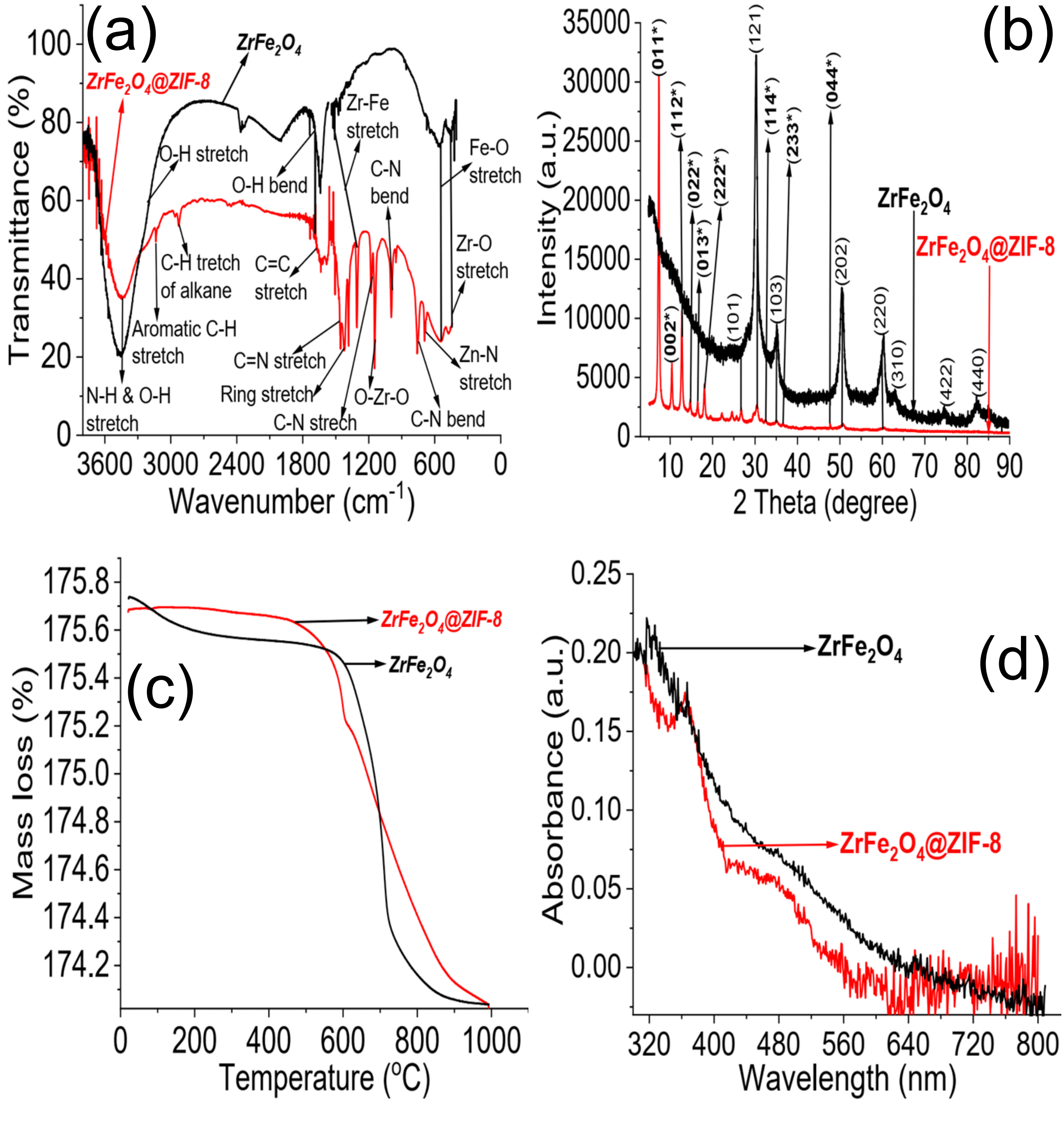

Fig. 1: FTIR (a), XRD (b), TGA (c) and UV-Visible spectra (d) of $ZrFe_2O_4$ and $ZrFe_2O_4$@ZIF-8

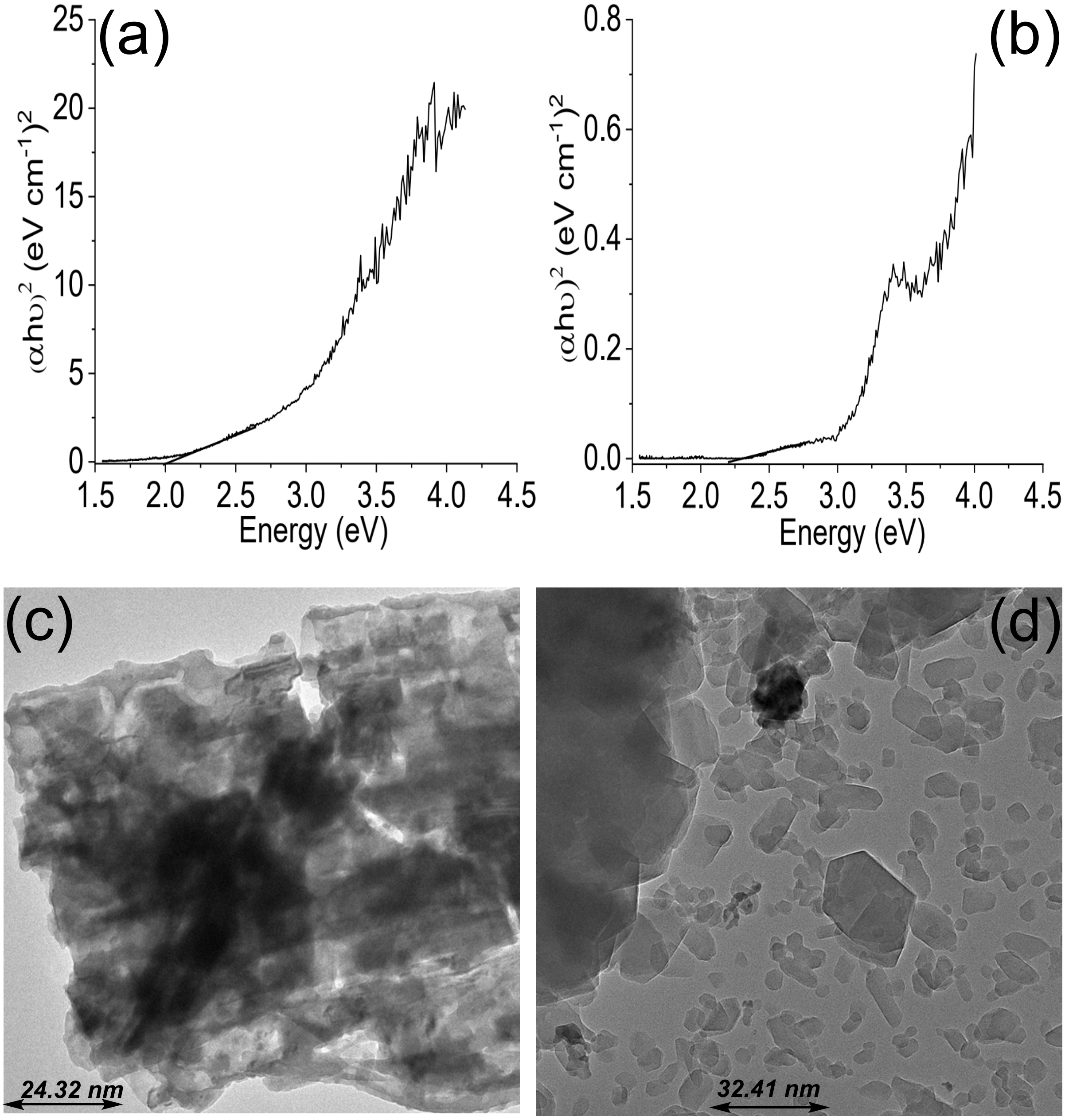

Fig. 2: Tauc's plot for $ZrFe_2O_4$ (a), Tauc's plot for $ZrFe_2O_4$@ZIF-8 (b), TEM of $ZrFe_2O_4$ (c) and TEM of $ZrFe_2O_4$@ZIF-8 (d)

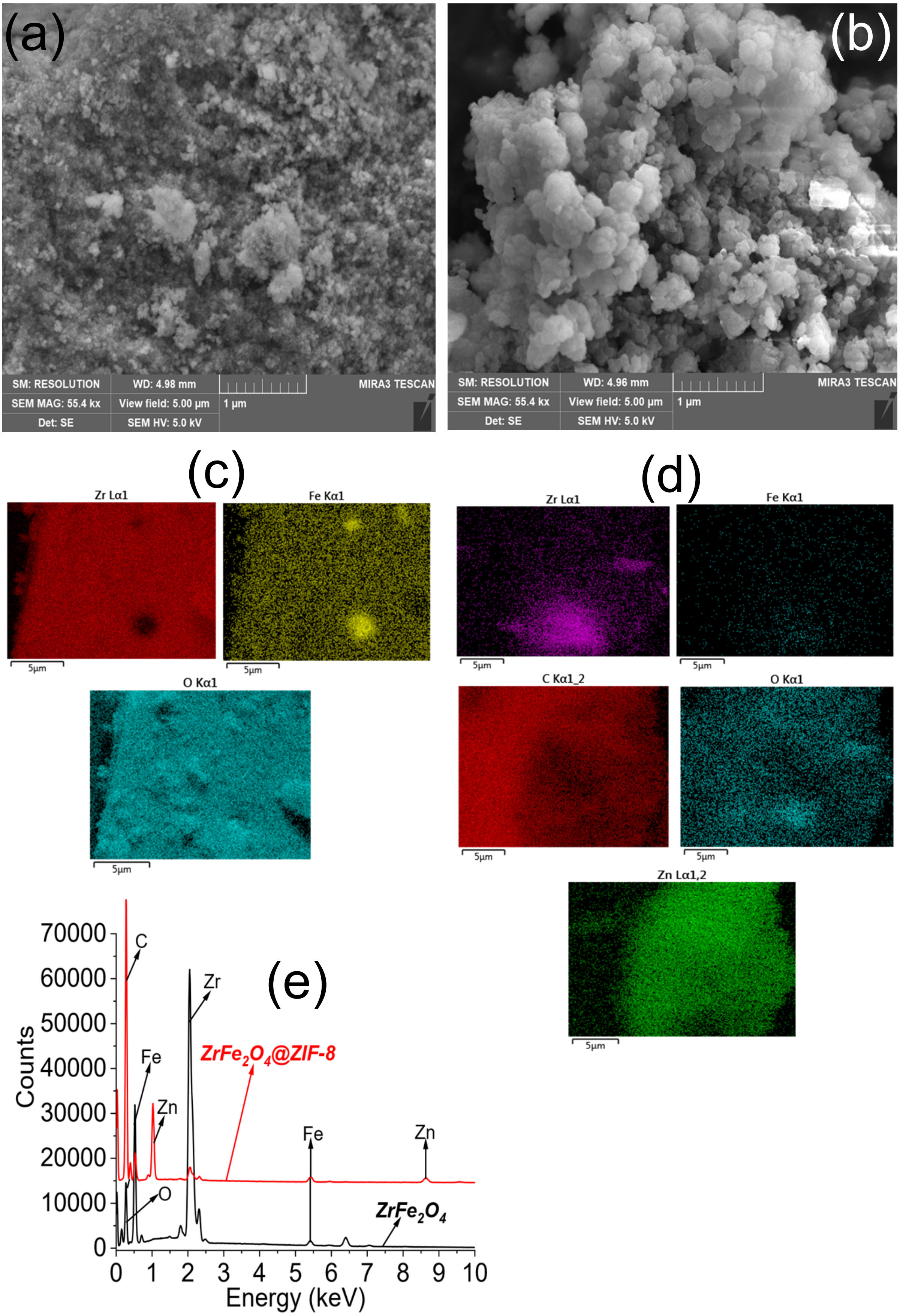
(a)
SM: RESOLUTION
WD: 4.98 mm
MIRA3 TESCAN
SEM MAG: 55.4 kx
View field: 5.00 µm
1 µm
Det: SE
SEM HV: 5.0 kV
(b)
SM: RESOLUTION
WD: 4.96 mm
MIRA3 TESCAN
SEM MAG: 55.4 kx
View field: 5.00 µm
1 µm
Det: SE
SEM HV: 5.0 kV
(c)
Zr Lα1
Fe Kα1
O Kα1
5µm
(d)
Zr Lα1
Fe Kα1
C Kα1_2
O Kα1
Zn Lα1,2
5µm
(e)
Counts
Energy (keV)
C
Zr
Fe
Zn
O
ZrFe2O4@ZIF-8
ZrFe2O4
0
10000
20000
30000
40000
50000
60000
70000
0 1 2 3 4 5 6 7 8 9 10

Fig. 3: SEM of $ZrFe_2O_4$ (a) SEM of $ZrFe_2O_4$@ZIF-8 (b), elemental mapping of $ZrFe_2O_4$ (c), elemental mapping of $ZrFe_2O_4$@ZIF-8 (d) and EDS of $ZrFe_2O_4$ and $ZrFe_2O_4$@ZIF-8 (e)

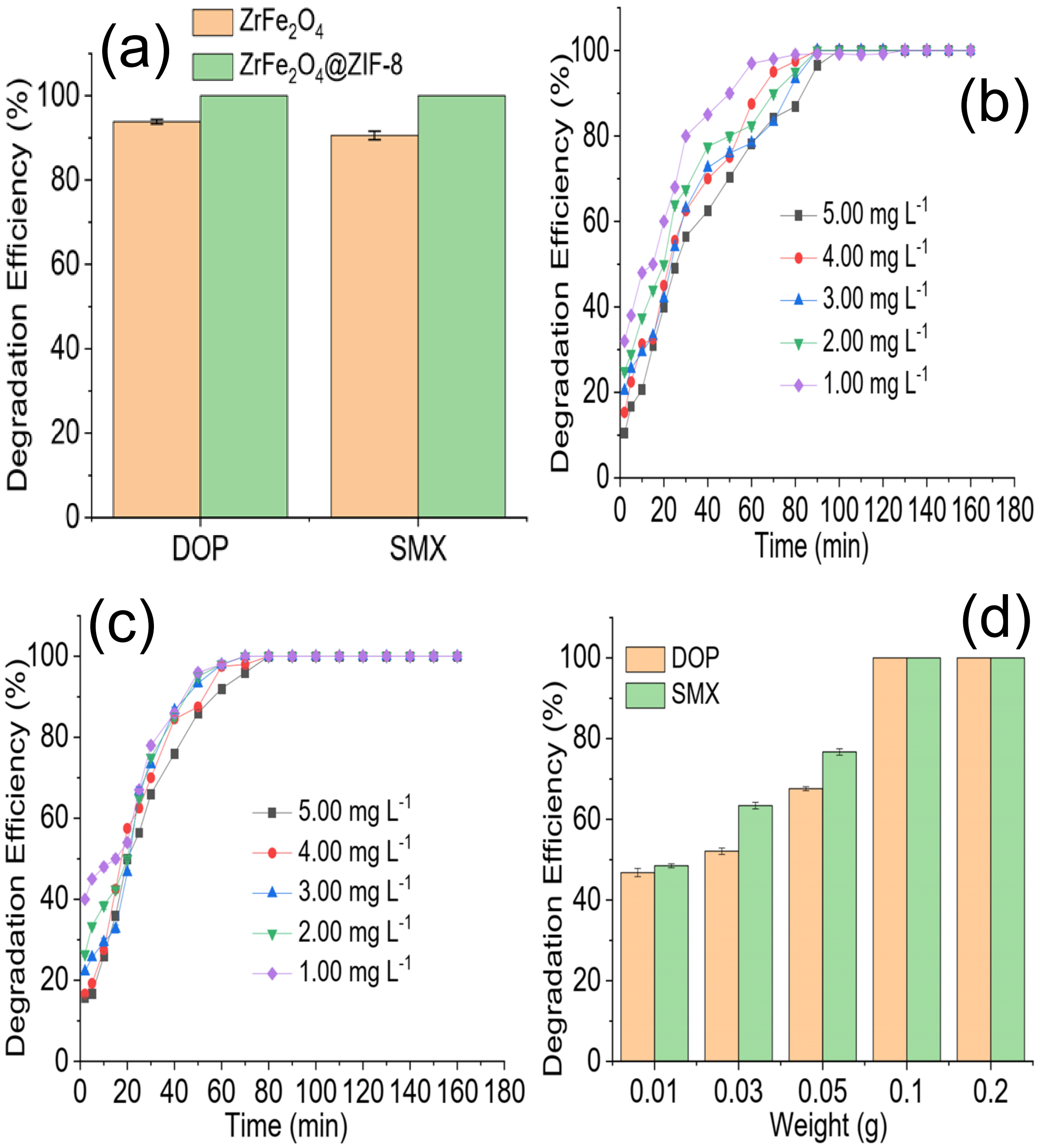

Fig. 4: Comparison of the preliminary degradation efficiency expressed by $ZrFe_2O_4$ and $ZrFe_2O_4$@ZIF-8 towards DOP and SMX (a), time dependent degradation of DOP in the presence of $ZrFe_2O_4$@ZIF-8 at different concentration (b), time dependent degradation of SMX in the presence of $ZrFe_2O_4$@ZIF-8 at different concentration (c) and effect of $ZrFe_2O_4$@ZIF-8 weight on the degradation of DOP and SMX (d)

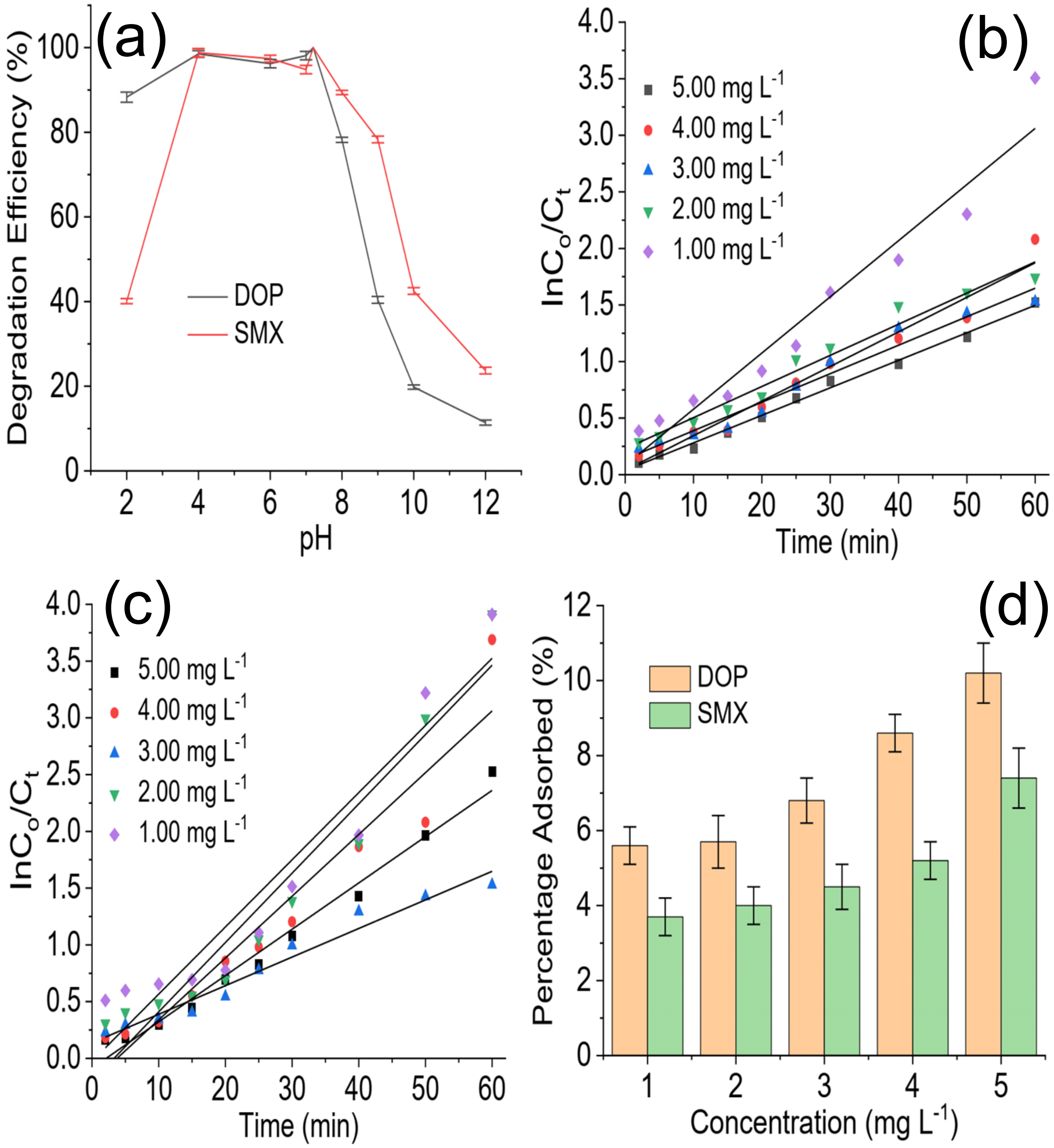

Fig. 5: Effect of solution pH on the degradation of DOP and SMX by $ZrFe_2O_4$@ZIF-8 (a), plot of $1nC_o/C_t$ versus irradiation time for the degradation of DOP (b) and SMX (c) at different solution concentrations in the presence of $ZrFe_2O_4$@ZIF-8 and percentage adsorbed during degradation of DOP and SMX by $ZrFe_2O_4$@ZIF-8 in the dark experiment (d)

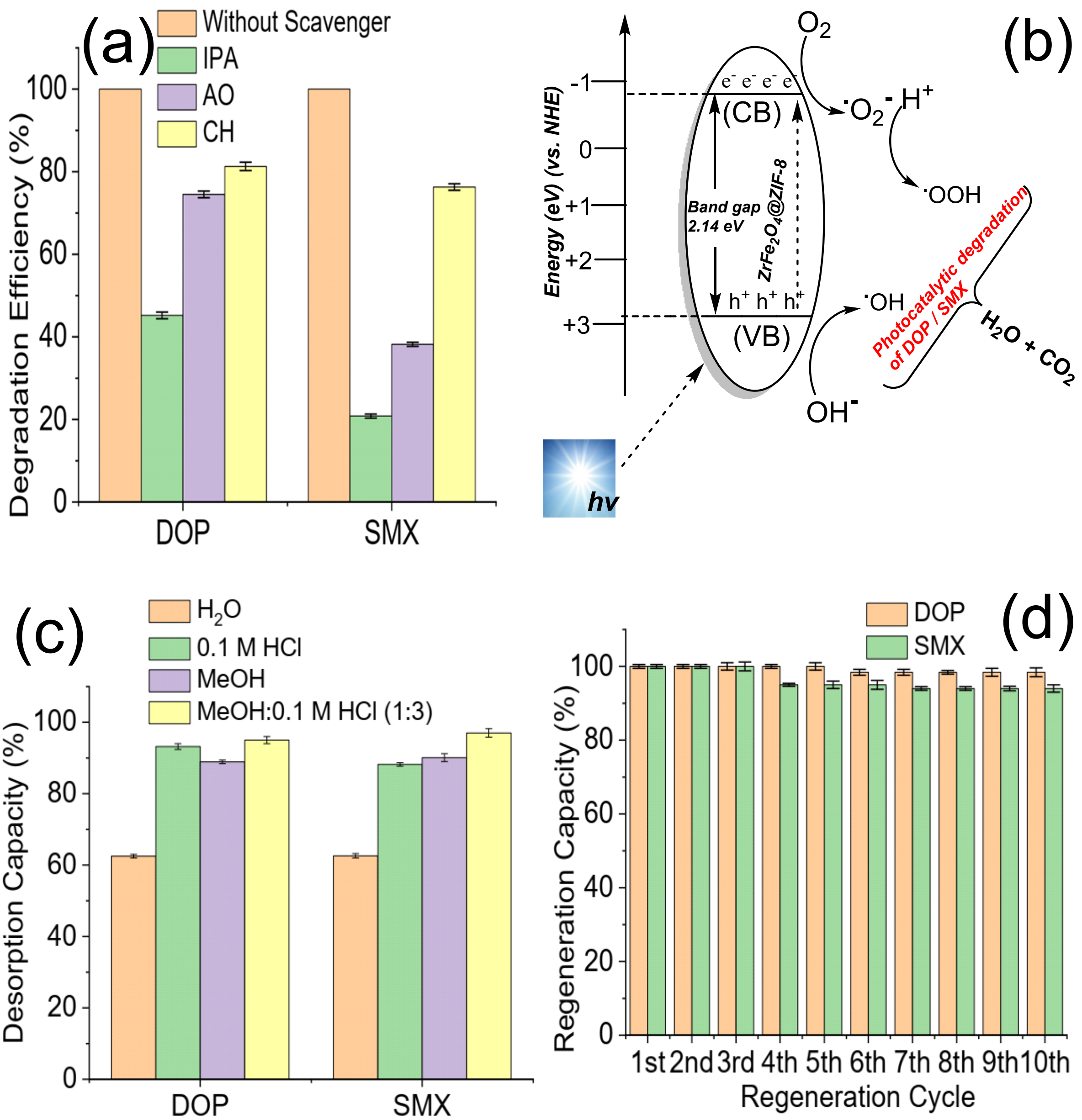

Fig. 6: Degradation efficiency of $ZrFe_2O_4$@ZIF-8 towards DOP and SMX with and without ROS scavengers (a), proposed mechanism for the photodegradation of DOP and SMX (b), desorption efficiency of $ZrFe_2O_4$@ZIF-8 after washing with different solvent systems (c) and regeneration capacity of $ZrFe_2O_4$@ZIF-8 expressed towards DOP and SMX at different treatment cycle (d)

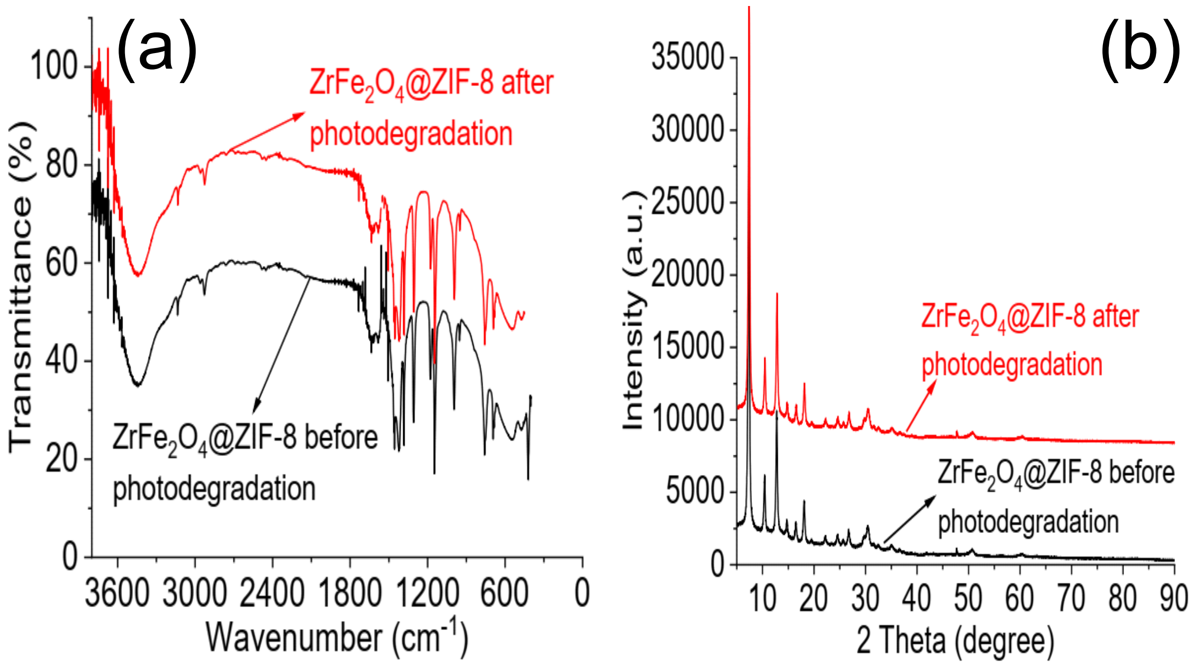

Fig. 7: FTIR of $ZrFe_2O_4$@ZIF-8 before photodegradation and at 10th cycle of photodegradation (a) and XRD of $ZrFe_2O_4$@ZIF-8 before photodegradation and at 10th cycle of photodegradation (b)
